# 3D Printed Devices for the Separation of Blood Plasma from Capillary Samples

**DOI:** 10.3390/mi15030359

**Published:** 2024-02-29

**Authors:** Giulia Deiana, Stewart Smith

**Affiliations:** School of Engineering, The University of Edinburgh, Scottish Microelectronics Centre, King’s Buildings, Alexander Crum Brown Road, Edinburgh EH9 3FF, UK; stewart.smith@ed.ac.uk

**Keywords:** 3D printing, sample preparation, point of care testing, microfiltration, cell separation

## Abstract

Sample preparation is a critical requirement for many clinical tests and diagnostic procedures, but it is difficult to perform on a lab-on-a-chip platform. The analytical side of microfluidic technologies has been gradually catching up with laboratory methods in terms of sensitivity, selectivity, and reliability. There is a growing need for the development of sample preparation modules that can either be connected or embedded into such devices and extract blood plasma in a fast, safe, and automated way. Achieving this functionality is an important step towards creating commercially viable products that can one day become part of everyday life. In this study, a range of simple, yet effective, 3D printed sample preparation devices was developed. The devices rely on snap-fit mechanisms and “resin-bonding” methods to fasten two layers and integrate a plasma separation membrane in between. The devices have excellent usability, with only one step required for their operation without any waiting time for the user, and could extract an average of 56.88% of the total available plasma from 50 μL capillary blood samples in 87 s without inducing any haemolysis. The manufacturing process is quick and straightforward, requiring only low-cost equipment and minimal training. The devices can either be used as a stand-alone device or integrated into an existing lab-on-a-chip system to provide blood filtration capabilities.

## 1. Introduction

Point-of-care (PoC) testing is a fast growing field with an increasing number of applications. The PoC market includes both over the counter products and professional devices used by practitioners and trained staff. Affordable point-of-care testing devices that can perform low-cost tests on the spot have the potential to simultaneously allow healthcare practitioners to provide early therapeutic intervention, and for patients to self-monitor chronic conditions. A classic example of this are glucose sensors, which have maintained their status as the most studied and widely available type of PoC device. Thanks to their fast turn-around times, low-cost, portability, user-friendliness and, most importantly, effectiveness, glucose monitors are now the standard method of care for diabetic patients worldwide [1]. The evolution of glucose monitors reflects the advances of PoC technologies over the decades. Along with glucose monitors, tests for infectious diseases are also a fast growing area due to the high demand in developing countries, where mortality due to epidemics is especially high and access to medical care is often insufficient [2]. Events such as the recent COVID-19 pandemic also boosted the demand for effective PoC testing devices and helped familiarise patients with rapid diagnostic technology.

As cellular material can interfere with analytical methods, many clinical tests and diagnostic procedures require the separation of blood cells from their liquid part (called plasma) prior to analysis. While this is easily achieved in clinical laboratories, replicating this functionality in miniaturised devices is notoriously difficult due to the complexity of whole blood as a non-Newtonian fluid with high viscosity and a tendency to coagulate rapidly if untreated [3]. In order to develop miniaturised lab-on-a-chip devices capable of performing clinical assays to laboratory standards, there is a growing need for sample preparation modules capable of extracting blood plasma in a fast, safe and automated way. When attached or embedded into sensing devices, such modules could help create full lab-on-a-chip platforms that can treat raw blood samples directly. This is an important step towards creating commercially viable products that will become part of everyday life.

The literature offers several examples of such devices based on a variety of techniques. Methods based on hydrodynamic effects [4,5,6], can be effective in a laboratory setting, but present significant issues in point-of-care settings where small capillary samples (≤100 μL) are used. Hydrodynamic filtering methods have relatively high blood volume requirements and low yield [4,7], as well as a frequent need for significant sample dilution and precise fluid control, which is difficult to achieve without expensive microfluidic infrastructure [6,8,9,10].

Microfiltration devices rely on either a separate or inbuilt porous membrane to physically separate plasma from cells. Depending on the configuration of the device and the properties of the membrane used, the input sample volume can go from a few tens of μL to ∼2 mL. Several devices based on microfiltration configurations can be found in the literature, featuring dead-end filtration [11,12,13,14,15], cross-flow filtration [16,17,18], sedimentation-assisted microfiltration [19,20,21] and immunological capture methods [22,23].

Many of the studies on microfiltration devices in the literature have been found to have limited data points and lack presentation of key parameters necessary to evaluate the quality of the separation process. For instance, in the study by Liu et al. [20], the authors did not mention the purity of haemoglobin level of the extracted plasma. Kadimisetty et al. [21] claimed a 94% plasma yield from their devices, but did not specify how the volume of the extracted plasma was measured and did not mention the haematocrit of the input sample. Furthermore, no information was given about the purity or the haemoglobin levels of the recovered plasma. In the study by Kim et al. [15], details on the original haematocrit level of samples and the purity and haemoglobin content in extracted plasma were not provided. Similarly, [14] provides no detail on plasma purity. Other studies, such as the one by Park et al. [24], did not include information on plasma yield or quality.

It is also worth noting that many of the microfiltration systems require complex and often manual procedures for fabrication and assembly, which can hinder their mass production, commercial viability, and usability in low-resource settings. Additionally, extraction times for these devices can typically exceed 5 min, sometimes even longer.

Other researchers have explored creating low-cost, hand-powered centrifuge systems using common household items such as egg beaters [25], salad spinners [26], or by mimicking a spinning paper toy [27]. While these attempts have shown some success, they may not be able to generate the same forces as commercial centrifuges, which could limit plasma extraction. Additionally, some designs may have higher forces but lack control over acceleration and deceleration, potentially leading to haemolysis. Similar to previous studies, the information provided regarding the original samples or the quality of the extracted plasma was incomplete.

This study explored sample preparation in its most simple form: a membrane placed between two 3D printed layers connected using integral mechanical attachments, which allow two parts to be joined without using any external component. Integral mechanical attachments, in the form of geometric features integrated in the mating parts, are used to create mechanical interference that prevents the parts from moving and/or separating. In plastic components, these are commonly referred to as “snap-fits”, due to the snap sound produced during the assembly of the mating parts. In this study, several snap-fit attachments were developed and compared, with the objective of both allowing the top and bottom layers to be fastened correctly and the edges of the membrane to be compressed to prevent blood from reaching and contaminating the separated plasma at the outlet. This method was compared to direct bonding of two device layers using the same resin used in the 3D printing of the devices, which achieved a complete seal around the edges of the device and the membrane to prevent any leaks. Microfiltration was selected as the main separation technique for its simplicity and compatibility with passive plasma extraction. Capillary samples with a volume of 50 μL were chosen for their ease of collection from finger pricks. Design-for-manufacturing and assembly principles were kept in mind during the design process so that the devices could potentially be mass-manufactured using standard techniques, such as injection-moulding, with only minor adaptations.

**Figure 1:**
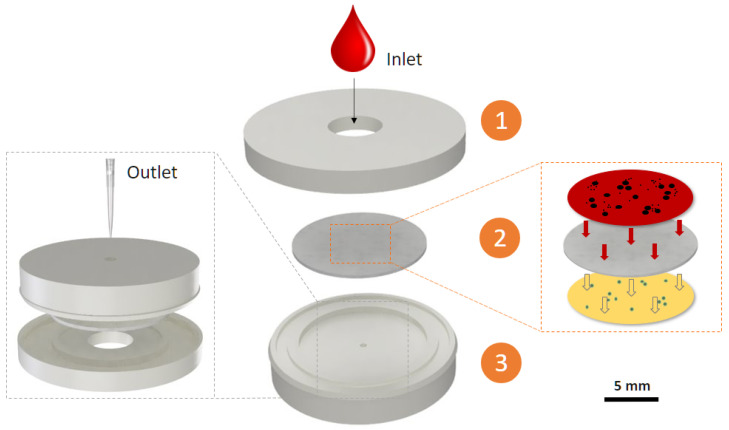
Schematic representation of the general structure and operational steps of the devices. Step 1: blood is deposited inside the device through the inlet. Step 2: the plasma is filtered by the membrane, which retains red blood cells and allows other blood components through for further analysis. Step 3: clear plasma is collected at the outlet using a pipette.

The plasma separation devices developed could extract a large amount of pure, high quality plasma within a few minutes while being easy to manufacture using low-cost 3D printing methods. They were easy to assemble and use by anyone without prior experience, with only a pipette being required for their operation as shown in Figure 1. These devices can help other researchers in furthering the field of point-of-care sensors requiring blood plasma separation by offering a simple, yet effective and highly integrable solution that can be used in the development of their devices.

## 2. Materials and Methods

### 2.1. Membrane Description

The membrane of choice throughout the project was the Vivid^TM^GR, an asymmetric polysulfone membrane developed by Pall Corporation, which is specifically engineered for blood plasma separation in human blood. The pore size at the upstream surface is approximately 100 μm and shrinks progressively to around 2 μm at the downstream surface [19]. This asymmetric structure helps trap red blood cells while preventing excessive haemolysis, leading to a higher quality plasma output compared to non-asymmetric counterparts. The membrane can handle human blood volumes up to 40–50 μL $cm^{-2}$ with a plasma recovery rate above 80% in under 2 min. A number of studies in the literature adopted this membrane with good results, confirming its capability to extract a high percentage of available plasma with low haemolysis and low protein binding.

The upstream part of this membrane is extremely hydrophilic, while the downstream side requires direct contact with a highly hydrophilic surface for liquid transfer to occur. As our material was not extremely hydrophilic, only partial transfer could be observed, and the plasma could only be successfully redirected through the outlet channel by obtaining of a pressure differential with a pipette. The question of how different membranes would behave when used within the devices is interesting, but not one we could answer in the timescale of this study. However, we expect to observe a similar principle of operation unless our material is somehow treated so that the surface can be made highly hydrophilic. Use of different pipettes was explored, and that specific one was selected as the speed of extraction was adequate to achieve high yield while limiting cell damage as the plasma passes through the membrane. The membrane was purchased in rectangular sheets directly from Pall Corporation and was cut to into circles of 13 mm diameter manually using a hollow hole punch set.

### 2.2. Device Manufacturing

The plasma separation devices were designed using Autodesk Fusion 360 and manufactured with the Original Prusa SL1 3D printer (Prusa Research) and the ELEGOO plant-based translucent photosensitive resin (purchased from ELEGOO via Amazon.co.uk). The parts were post-processed by thoroughly washing them in a 99% Isopropyl Alcohol bath to remove resin residuals before curing them under UV light using the Original Prusa Curing and Washing Machine (Prusa Research, Prague, Czech Republic). Internal channels were flushed manually with IPA and a 3 mL pastette (Alpha Laboratories, Easleigh, UK) prior to curing to prevent trapped resin from clogging the channels. As the resin is still soft immediately after extraction from the 3D printer, this method proved very effective in removing residual resin from the short internal channels of the devices. Occasionally, the prints at the bottom edge would become overcured due to the imperfect mechanism of operation of the 3D printer model used. In this case, the resin could easily be scraped off using a small and sharp object, and the channel could then be flushed normally using IPA. Visually, a device was considered ready when IPA could clearly be seen exiting the channel at speed during flushing. The use of fresh IPA is highly recommended to obtain the best cleaning results.

### 2.3. Design and Assembly

In order to determine the best design parameters, the limits of the 3D printer used were explored using a range of test prints. This allowed us to establish the minimum channel diameter and its ideal shape. A description of such prints and our findings can be found in the Supplementary Materials. All devices have an internal channel diameter of 0.7 mm, which is 0.1 mm wider than our 3D printer’s limit, which guarantees a good, consistent result after every print. Lowering the diameter resulted in channels that either clog during printing, or immediately after removal from the 3D printer, in such a way as to make the devices unusable.

**Figure 2:**
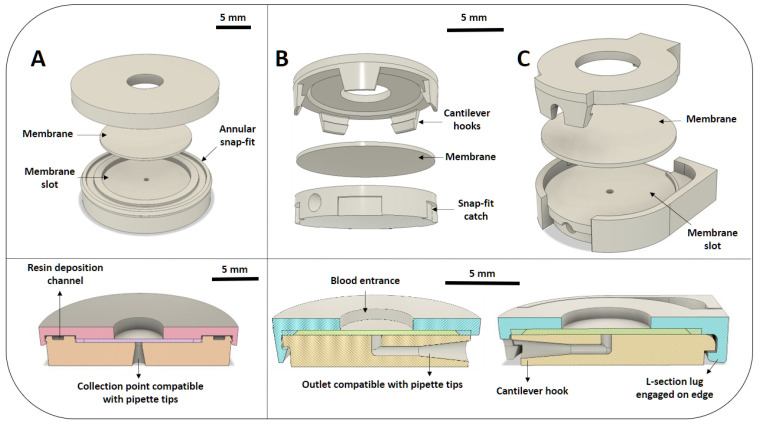
(**A**) Exploded view and cross-section of the final prototype of bonded 3D printed devices. Highlighted are the resin deposition channel, where uncured resin is deposited prior to assembly and bonding, and the annular snap-fit holding the device together before bonding and the membrane clamp. (**B**) Exploded view and cross section of the multiple hook snap-fit devices. When engaged, the cantilever hooks hold the top and bottom parts together, while compressing the edges of the membrane to prevent spills towards the outlet. (**C**) Exploded view and cross section of single hook snap-fit devices. The inflexible locator offers more stability than the configuration with only cantilever hooks, meaning that the membrane can be better compressed at the edges. Having only one locking feature, these devices are also easier to assemble and disassemble than the multi-hook version. A detailed overview of the device dimensions and offsets used for 3D printing can be found in the Supplementary Materials file. This also includes links to the designs in Autodesk Fusion 360 which are publicly available.

**Figure 3:**
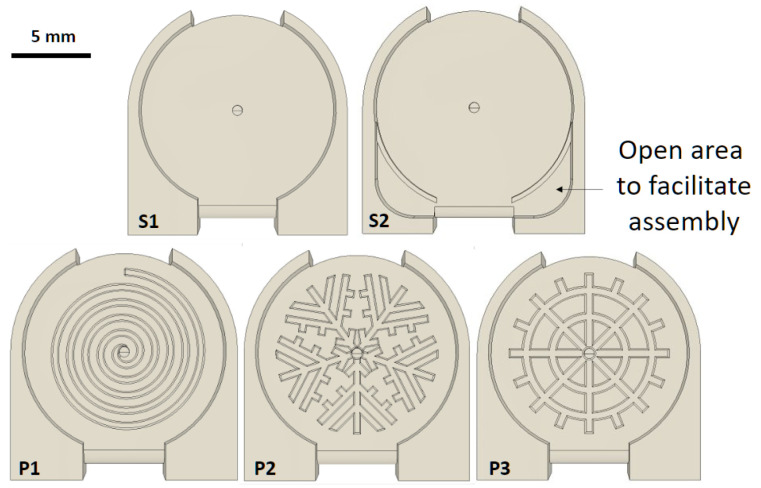
Labelled bottom layer of devices used. While the P1 and P2 patterns are original, the P3 pattern was adapted from the work of Kadimisetty et al. [21], where it was claimed to have significantly improved the yield of their devices.

All devices comprised top and bottom layers either bonded together by photopolymerisation of the same resin used during the printing process (B), or attached through a snap-fit connection. The snap-fit devices are further divided into those having single hook (S) and multiple hooks (M), as shown in Figure 2. The two different types of single hook devices (S1 and S2) differ for the presence of an open area in S2 that facilitates the assembly process, as shown in Figure 3.

The layers of the devices were 3D printed flat against the surface of the print platform. This greatly reduced both the printing time and material consumption as opposed to the classic tilted orientation with pads and supports. The features in each design were carefully planned to remain within the capabilities of the printer, particularly with regards to overhangs, tolerances/clearances between features and supports.

**Figure 4:**
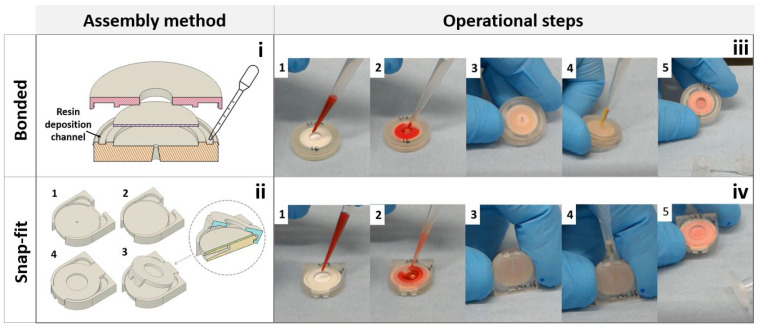
Assembly methods and operational steps of bonded and snap-fit devices. Although the single hook S2 devices were used for illustrative purposes, all snap-fit devices were operated in the same way. (**i**) Schematic representation of the resin deposition process. During the manufacturing process, a pastette is used to fill the resin deposition channel with raw resin prior to assembly of the top and bottom layer. (**ii**) Lock mechanisms developed for the single hook snap-fit devices. The numbers indicate the assembly steps. In step 1, the bottom layer of the device is shown as the device is prepared for assembly. In step 2 the membrane is added, while in step 3 the cap is assembled by pivoting the lug at the top layer around the locator in the bottom layer. Inset image offers a view of the cross section of the device during step 3. In step 4 the device is shown fully assembled. (**iii**) Operational steps of the 3D printed bonded devices. (1) The assembled device is ready to be used. (2) Blood is placed inside the device. (3) The device is flipped upside-down to place the outlet in a favourable position for extraction. (4) The plasma is collected immediately using a 100 μL pipette and transferred inside a centrifuge tube for further assessment. (5) The device can be safely discarded. (**iv**) Operational steps of the 3D printed snap-fit devices. (1) The assembled device is ready to be used. (2) Blood is placed inside the device. (3) The device is placed on its side to expose the outlet and facilitate the extraction. (4) The plasma is collected immediately using a 100 μL pipette and transferred inside a centrifuge tube for further assessment. (5) The device can be safely discarded.

The “bonded” devices were assembled as follows:

1. The membrane was placed on the bottom layer and a small amount of resin was pipetted within the 1.15 mm wide and 0.4 mm deep resin deposition channel shown in Figure 4i.
2. The top and bottom were then joined and held together through the annular snap-fit. The 0.8 mm wide and 0.8 mm deep membrane clamp provided a barrier that helped prevent the resin from reaching and soaking the membrane, while also preventing blood from freely flowing around the edges of the membrane during testing.
3. The assembled devices were then cured under UV light for 10 min on each side, after which they were ready to use.

In the bonded devices, the diameter of outlet was sharply tapered to block the pipette tip before it could reach, and therefore damage, the membrane. Due to the small printing area of the 3D printer used, with a maximum printing volume of 120×68×150 mm, up to 8 “bonded” devices could be fabricated in one batch with a total fabrication time of 50 min. As snap-fit devices were smaller and consumed less material than the bonded version, up to 15 could be fabricated in one batch in 45 min. Another clear advantage of this device type was the simplified assembly procedure:

- The membrane was placed in its slot in the bottom layer, as shown in Figure 2.
- Multiple hook devices: the cantilever hooks in the top layer were aligned with the catches in the bottom layer and the two were then joined as uniformly as possible until all cantilevers were engaged against their respective catch.
- Single-hook devices: the top layer was assembled by first aligning the lug under the locator feature, then pivoting the cap and finally snapping the cantilever hook to the lock in the front of the device (the procedure is illustrated in Figure 4ii).

In the snap-fit devices, the outer edge of the top layer (0.7 mm wide and 0.4 mm deep) acted as the membrane clamp to compress the edge of the membrane and prevent blood from reaching the outlet area.

The outlets of both bonded and snap-fit devices were shaped to accommodate 300 μL pipette tips. The features in all types of patterned devices were 0.5 mm wide and 0.1 mm deep.

### 2.4. Cost Analysis of Manufacturing and Use

The equipment and materials required for the fabrication and assembly of the devices were relatively inexpensive. We purchased the Original Prusa SL1 kit + Curing and Washing Machine bundle in 2020 (now discontinued) for £1329. However, there are now equivalent 3D printers from manufacturers such as ELEGOO and ANYCUBIC that can be bought for as little as £350. The ELEGOO plant-based used throughout this study could be purchased for £30 per litre and the resin cost per device was as low as £ 0.05.

Each membrane sheet from Pall Corporation was purchased for £19.82 and a total of 234 membranes could be obtained from each sheet, meaning that the cost per membrane was £0.08. Although we used Eppendorf pipettes throughout this study, any equivalent low-cost pipette can be used with the same effect. The affordable and accessible hobbyist equipment necessary for the fabrication and use of these devices makes them accessible to many research groups, who are likely to already have similar resources at their disposal.

### 2.5. Device Testing and Haemoglobin Measurement

Equine blood samples used in the experiments were obtained from the Easter Bush Pathology Laboratory (Royal (Dick) School of Veterinary Studies, The University of Edinburgh). The laboratory was unable to provide date and time of extraction for the samples. As such, the age and quality varied significantly for each specimen used in the experiments. All samples were treated with Ethylenediaminetetraacetic acid (EDTA) and were used within 24 h of arrival at the Pathology Laboratory. The plasma purity was assessed using the Neubauer-improved haemocytometer (Marienfields), which was used according to the manufacturer’s instructions.

The steps necessary to operate the devices are illustrated and explained in Figure 4iii,iv. While most of the procedure is similar, the bonded devices are operated either flat or at a slight angle, while the snap-fit devices are placed vertically on one side with the extraction port uppermost. In both cases, the extraction is quick and simple, as the plasma could be extracted directly with a pipette immediately after blood insertion without any additional steps or waiting time for the user.

Once the plasma was extracted, its volume was measured with a set of calibrated Eppendorf Research Plus pipettes. The haemoglobin concentration was then calculated directly from the spectrum of the plasma sample using the method first described by Allen [28] and then Cripps [29]. Briefly, each sample was thoroughly mixed and its spectrum measured using the NanoDrop 1000 (Thermo Scientific). Cripps’ H-value was then calculated and fitted to a standard curve generated specifically for this study to obtain the corresponding haemoglobin value.

To prepare the control plasma, the sample was thoroughly mixed for 30 s by inversion. Then, 50 μL of blood was pipetted into a 2 mL tube and spun at 1600× *g* for 10 min in an Eppendorf 5418 R centrifuge. The supernatant was transferred into a clean tube and spun again in the same centrifuge at 12,000× *g* for 10 min. Finally, the plasma was transferred into a clean tube and its haemoglobin concentration measured.

## 3. Results and Discussion

### 3.1. Principle of Operation

Upon placing a blood droplet on the membrane, the plasma separated accumulates at the interface between the downstream part of the membrane and the surface of the device. To drive the plasma through the outlet and into a collection medium, suction was applied at the outlet using a pipette.

The flow of plasma within the device was laminar. This was calculated using the Reynolds Number calculation for flow in a pipe:

(1) $$Re=\frac{uD_{H}}{v}$$

where *u* is the fluid velocity ($m\;s^{-1}$), $D_{H}$ is the inner diameter of a circular pipe (m) and *v* is the kinematic viscosity. The kinematic viscosity can be calculated as follows:

(2) $$v=\frac{\mu }{\rho }\;\left(m^{2}\;s^{-1}\right)$$

where μ is the dynamic viscosity and ρ is the density of the substance. For practical purposes the temperature dependency will be ignored in this calculation, as the experiments were carried out at room temperature. The normal dynamic viscosity of blood plasma at $37^{\circ }C$ is ∼0.00125 Pas [30] while plasma density is dependent on sample haematocrit and on average estimated to be 1025 kg $m^{-3}$ [31]. This means that the kinematic viscosity $v\approx 1.22\times 10^{-6}\;m^{2}s^{-1}$. The inner diameter of the channel was 0.7 mm and its length 6.5 mm. As it was filled in 1 s during the experiments, the velocity is $0.0065\;m\;s^{-1}$. With this information, the Reynolds Number can be calculated as 3.731, which is well below the threshold for turbulent flow.

Now that the flow has been demonstrated to be laminar, the Hagen-Poiseuille equation for laminar flow in a channel of circular cross section can be used to describe the effect of the pressure applied on the plasma flow within the device:

(3) $$\Delta p=\frac{8\mu QL}{\pi R^{4}}$$

where Δp is the pressure difference between inlet and outlet, *Q* is the volumetric flow rate, *L* is the pipe length, *R* is the channel radius and μ is the dynamic viscosity of the fluid passing through the channel.

In Equation (3), Δp is directly proportional to the channel length, meaning that a larger pressure difference is needed to move the same fluid volume at the same rate along a longer pipe. As such, the channel length was minimised in the design to maximise the potential yield, while still creating an effective barrier for the pipette tip so that the membrane surface would not be damaged during extraction.

Using a more hydrophilic material would likely allow for a more efficient extraction. The objective of this study, however, was to analyse the performance of the cheapest type of resins available for low-cost 3D printing. As such we did not explore any other material using the designs proposed in this work.

### 3.2. Analysis Parameters

The following parameters will be used to describe the performance of the devices developed in this study:

- *Yield*: refers to the percentage of available plasma recovered by the device.
- *Plasma quality* or *Hgb difference*: the lower the haemolysis induced by the devices during extraction, the higher the quality of the extracted plasma. The devices were tested with a wide range of small samples, each having different age, quality and haematocrit levels. To remove the effects of initial sample quality from the device analysis, the plasma quality was quantified as Hgb_device_ − Hgb_control_, that is the difference between the haemoglobin concentration of the plasma extracted with the devices and the corresponding control plasma. The lower this value, the better the plasma quality extracted from the device as it means that no additional haemolysis was caused by the extraction process.
- The *extraction time* indicates the time necessary for the complete extraction process from the moment the sample is placed inside the device.
- The *failure rate* refers to the percentage of devices that failed completely during testing. A device is considered to have failed when an excessive amount of input blood makes its way from the upstream to the downstream area of the membrane, as a consequence of either membrane damage or insufficient compression at the membrane edges. It is important to note that some of the membrane sheets used were damaged by transportation or while being cut by hand before being incorporated into the device during the assembly process. Obviously damaged membranes were immediately discarded and utmost care was taken during the manufacturing process. Despite this, an unknown percentage of devices was fitted with damaged membranes, therefore leading to failure. Although it was not possible in this study, this issue can be avoided by using new and damage-free membrane sheets prepared with other methods, such as laser cutting.
- The *visible RBCs rate* refers to the percentage of devices that allowed a visible, but small amount of residual cells in the output plasma. Plasma with minimal visible cell residual still maintained a purity >99.9% as measured by the haemocytometer. Because of this, devices with minimal residual cells were treated as successful and used as a valid data point for analysis.

### 3.3. Device Characterisation

**Figure 5:**
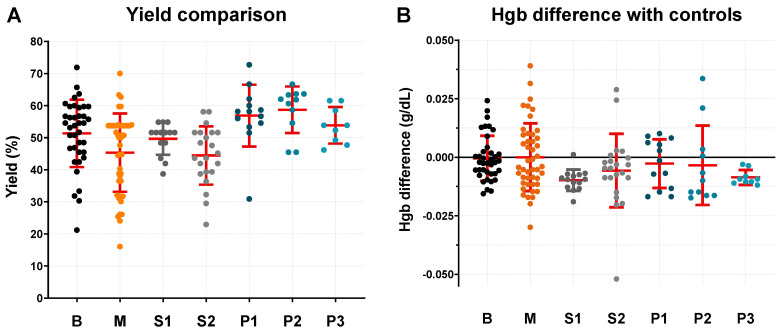
Yield (**A**) and plasma quality (**B**) comparison between all iterations of 3D printed bonded devices. Plasma quality was measured as haemoglobin concentration difference with centrifuged controls, rather than actual Hgb concentration, due to differences in Hgb content between different samples used for testing. The red lines represent population mean and standard deviation.

A summary of all data collected can be found in Table 1, while Figure 5 shows the data points gathered for all the different types of 3D printed devices with regards to yield and difference in Hgb concentration with centrifuged controls. All device types achieved a yield >44% and a Hgb concentration almost identical to controls, confirming that, when operated correctly, all types of these simple plasma separation devices are able to extract a good percentage of high quality plasma.

Out of all the non-patterned devices (B, M, S1 and S2), the bonded type (B) presented the highest average yield (51.34%). The S1 devices also obtained a similar yield (49.65%) while simultaneously achieving the best overall plasma quality, with a measured Hgb concentration on average 0.0098 $g\;dL^{-1}$ lower than centrifuged controls. The extraction time was significantly higher for the multi-hook devices, with an average of 166.75 s necessary for a successful extraction against 76.11 and 97.92 s for the bonded and single hook devices respectively. The multi-hook devices also presented a much higher failure rate (14.55%) and were more difficult to assemble during the manufacturing process, making them the least appealing type out of those tested.

Although the extraction time and failure rate of the single hook devices are overall slightly higher than the bonded devices, the single hook devices have a far easier manufacturing process thanks to their intuitive and robust snap-fit mechanism. Out of the two single hook designs proposed (S1 and S2 as shown in Figure 3), the S2 devices are simpler to assemble, but obtained a significantly lower yield and higher failure rate. As such, they can only be recommended as a valid alternative to S1 and bonded devices in scenarios where assembly time and costs need to be reduced.

### 3.4. Experimental Observations

The lower yield and higher extraction time of the single and multi hook device can be explained as a consequence of a weaker closing mechanism. While in the bonded devices the membrane edges are firmly enclosed by hardened resin, the snap-fit devices relies on the strength of the mechanical interference between interlocked device features to create pressure on the edge of the membrane. The more the interference, the stronger the “bond” and the resulting clamping force on the membrane’s edge. However, higher interference makes the assembly of the devices more difficult.

In this study, we attempted to come as close as possible to a trade-off between mechanism strength and ease of assembly by hand. Our easiest-to-assemble devices, the S2 type, and the M devices are both affected by low yield, high extraction time and a higher failure rate, which is indicative of a weaker clamping mechanism on the membrane’s edges. This increases the likelihood of air and blood being pulled from the edges of the membrane instead of the wanted outcome, which is for the plasma accumulated on the downstream part of the membrane to be extracted through the outlet.

In an effort to address these challenges, three different patterns for the plasma collection area in contact with the downstream side of the membrane were tested, with the aim of facilitating the plasma extraction process and potentially obtaining a higher volume of good quality plasma. The patterns are shown in Figure 3 along with their respective identifying code. The introduction of a pattern led to significant improvements in yield and extraction time. However, both P2 and P3 led to a noticeable increase in the failure rate of the devices.

### 3.5. Anti-BRSV Ab ELISA

**Figure 6:**
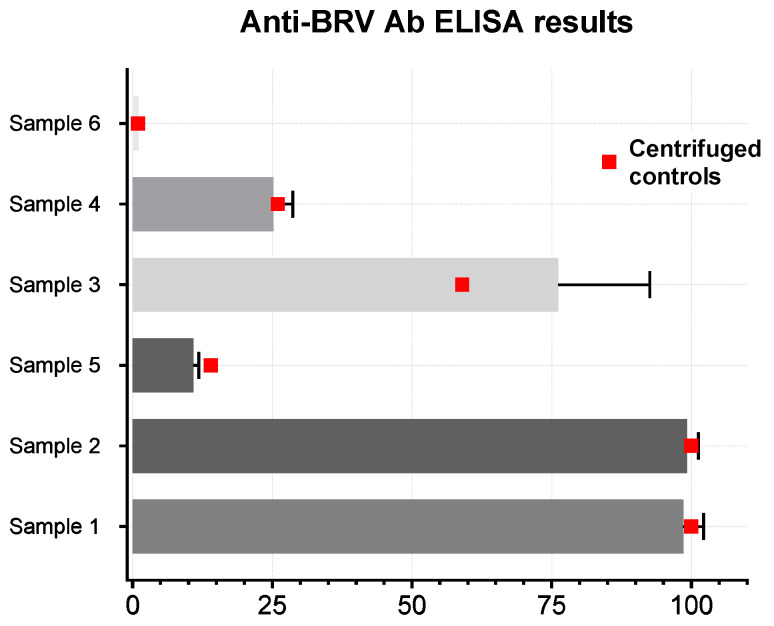
Comparison of ELISA results of centrifuged control against 6 devices for 6 different samples for a total of 36 devices tested. Each column represents the average of the 6 data points collected. Each data point was calculated by fitting the absorption measured from the sample and assay solutions prepared as per assay protocol on a 96 wells plate to an 8 point standard curve to determine the concentration of antibodies present in the sample. Results >3 indicate antibody presence.

The plasma produced by the bonded 3D printed devices was tested with a two-step competitive enzyme-linked immunosorbent assay (ELISA) for the detection of antibodies to the Bovine Respiratory Syncytial Virus (BRSV), which infects the respiratory tract of cows resulting in a mild to life-threatening respiratory disease. Antibodies to this virus are commonly found in local cattle. The testing was outsourced to a local company (Biobest Laboratories Ltd., https://biobest.co.uk/). Six bovine samples were used to test 36 plasma separation devices, for a total of six devices used with each sample. The results are summarised in Figure 6.

Other than a few inconsistencies due to inter-test variability (as confirmed by the provider), the results from the devices closely matched those of the centrifuged control, meaning that the extracted plasma can be safely used in immunoassays.

### 3.6. Re-Usability Analysis

Although reusing devices previously contaminated with blood is discouraged, when other biological input matrices are used there is a potential to disinfect and reuse this type of device to reduce laboratory waste. To determine whether it was possible to safely and successfully reuse the snap-fit devices, some S2 devices were used and then disassembled, cleaned and disinfected using the following procedure:

1. Device parts that still contained blood after disassembly were scrubbed with 70% Ethanol and a toothbrush.
2. All parts were placed inside a waterproof container and soaked for 15 min in a TriGene ADVANCE disinfectant solution.
3. The parts were then rinsed 3× times in tap water, scattered onto an absorbent tissue and left to dry completely.

The devices were tested up to two times. Although the yield was unaffected, the failure rate increased dramatically after each use and the plasma quality decreased, suggesting that the disinfection protocol (standard in our laboratories for blood contaminated surfaces) caused material damage to the snap-fit mechanism, thus preventing it from locking the two layers together as effectively as during the first use.

## 4. Conclusions

**Figure 7:**
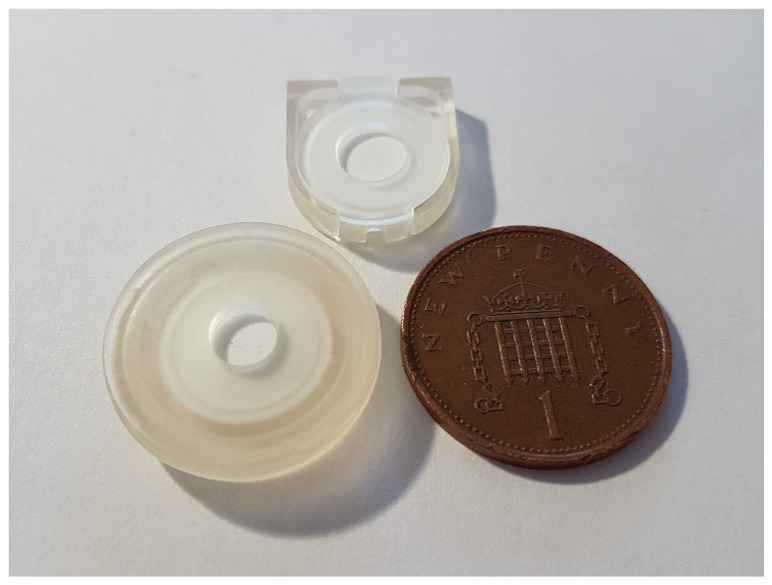
Assembled single hook device placed next to a bonded 3D printed device and a one penny coin. The snap-fit device is smaller and less material-consuming than its bonded counterpart.

This study analysed the performance and manufacturability of the simplest type of microfiltration device for blood plasma separation. The devices, shown in in Figure 7, are low-cost, small and material-efficient. They also required minimal equipment for their operation and were easy to fabricate, assemble and use.

Unlike most other studies, a large number of these devices was tested to determine their capabilities. Despite their simplicity, these 3D printed plasma separation devices outperform most competition in the literature, with the most successful device type being able to collect an average of 56.88% of the available plasma without inducing significant haemolysis during the separation process. This is indicated by the minimal haemoglobin concentration difference with control plasma samples prepared using standard centrifugation techniques. Moreover, the equipment necessary for the fabrication of the 3D printed devices described here is accessible both in terms of cost and simplicity of usage, thus greatly reducing the cost and effort required for the manufacturing process. The plasma extracted was used to successfully perform an immunoassay for the detection of antibodies to BRSV, with results closely matching those from centrifuged plasma controls. This indicates low protein loss within the devices, as previously confirmed by other studies in the literature that used similar microfiltration membranes and materials.

Finally, the snap-fit 3D printed devices are more eco-friendly than most alternatives, as they were produced using a plant-based biodegradable resin and can potentially be disassembled, disinfected and re-used. If 3D printed with high temperature-resistant resins, the devices can be easily autoclaved, which is the standard sterilisation procedure in most laboratories. The development of these plasma separation devices can hopefully contribute towards the creation of point-of-care devices or lab-on-a-chip components that require pure plasma for their analyses.

## Figures and Tables

**Table 1:** Data analysis summary of the 3D printed devices. The yield refers to the percentage of total available plasma extracted. The Hgb difference was calculated by subtracting the measured Hgb concentration of each device from a centrifuged control. The extraction time was calculated from blood sample insertion to complete plasma collection. Failure rate % refers to the percentage of the total devices tested that failed to produce any measurable volume of plasma or that experienced a significant amount of blood leakage towards the outlet. The visible RBCs % describes the percentage of the total devices tested that produced plasma with a small quantity of visible RBCs.

	Average			Standard Deviation			Other Info		
Device Type	Yield (%)	Hgb Diff. (g dL^−1^)	Extraction Time (s)	Yield (%)	Hgb Diff. (g dL^−1^)	Extraction Time (s)	Failure Rate (%)	Visible RBCs (%)	Number Tested
**(B) Bonded**	51.34	−0.0005	76.11	10.35	0.0095	14.82	4.65	9.30	43
**(M) Multi-hook**	45.35	<0.0001	166.75	12.08	0.0143	0.72	14.55	1.82	55
**(S1) Single-hook V1**	49.65	−0.0098	93.43	4.81	0.0044	17.15	0.00	0.00	14
**(S2) Single-hook V2**	44.45	−0.0057	100.77	8.87	0.0153	24.95	11.11	14.81	27
**(P1) S1 + Pattern 1**	56.88	−0.0027	86.77	9.25	0.0100	12.31	0.00	7.14	14
**(P2) S1 + Pattern 2**	58.71	−0.0034	84.27	6.92	0.0162	13.50	14.29	35.71	14
**(P3) S1 + Pattern 3**	53.85	−0.0086	80.67	5.38	0.0030	4.78	30.77	0.00	13

## Data Availability

The data presented in this study are openly available in the University of Edinburgh’s Data Share repository at https://doi.org/10.7488/ds/7695.

## Supplementary Materials

The following supporting information can be downloaded at: https://www.mdpi.com/article/10.3390/mi15030359/s1.

## References

[1] Teymourian H., Barfidokht A., Wang J. (2020). Electrochemical Glucose Sensors in Diabetes Management: An Updated Review (2010–2020). Chem. Soc. Rev..

[2] Shu T., Hunter H., Zhou Z., Sun Y., Cheng X., Ma J., Su L., Zhang X., Serpe M.J. (2021). Portable Point-of-Care Diagnostic Devices: An Updated Review. Anal. Methods.

[3] Kersaudy-Kerhoas M., Sollier E. (2013). Micro-Scale Blood Plasma Separation: From Acoustophoresis to Egg-Beaters. Lab Chip.

[4] Tripathi S., Varun Kumar Y.V.B., Prabhakar A., Joshi S.S., Agrawal A. (2015). Performance Study of Microfluidic Devices for Blood Plasma Separation—A Designer’s Perspective. J. Micromech. Microeng..

[5] Tripathi S., Kumar Y.V.B., Agrawal A., Prabhakar A., Joshi S.S. (2016). Microdevice for Plasma Separation from Whole Human Blood Using Bio-Physical and Geometrical Effects. Sci. Rep..

[6] Prabhakar A., Kumar Y.V.B.V., Tripathi S., Agrawal A. (2015). A Novel, Compact and Efficient Microchannel Arrangement with Multiple Hydrodynamic Effects for Blood Plasma Separation. Microfluid. Nanofluid..

[7] Yang S., Ündar A., Zahn J.D. (2006). A Microfluidic Device for Continuous, Real Time Blood Plasma Separation. Lab Chip.

[8] Kersaudy-Kerhoas M., Kavanagh D.M., Dhariwal R.S., Campbell C.J., Desmulliez M.P.Y. (2010). Validation of a Blood Plasma Separation System by Biomarker Detection. Lab Chip.

[9] Rafeie M., Zhang J., Asadnia M., Li W., Warkiani M.E. (2016). Multiplexing Slanted Spiral Microchannels for Ultra-Fast Blood Plasma Separation. Lab Chip.

[10] Robinson M., Marks H., Hinsdale T., Maitland K., Coté G. (2017). Rapid Isolation of Blood Plasma Using a Cascaded Inertial Microfluidic Device. Biomicrofluidics.

[11] Thorslund S., Klett O., Nikolajeff F., Markides K., Bergquist J. (2006). A Hybrid Poly(Dimethylsiloxane) Microsystem for on-Chip Whole Blood Filtration Optimized for Steroid Screening. Biomed. Microdevices.

[12] Homsy A., van der Wal P.D., Doll W., Schaller R., Korsatko S., Ratzer M., Ellmerer M., Pieber T.R., Nicol A., de Rooij N.F. (2012). Development and Validation of a Low Cost Blood Filtration Element Separating Plasma from Undiluted Whole Blood. Biomicrofluidics.

[13] Hauser J., Lenk G., Hansson J., Beck O., Stemme G., Roxhed N. (2018). High-Yield Passive Plasma Filtration from Human Finger Prick Blood. Anal. Chem..

[14] Gao Q., Chang Y., Deng Q., You H. (2020). A Simple and Rapid Method for Blood Plasma Separation Driven by Capillary Force with an Application in Protein Detection. Anal. Methods.

[15] Kim J., Yoon J., Byun J.Y., Kim H., Han S., Kim J., Lee J.H., Jo H.S., Chung S. (2021). Nano-Interstice Driven Powerless Blood Plasma Extraction in a Membrane Filter Integrated Microfluidic Device. Sensors.

[16] Aota A., Takahashi S., Mawatari K., Tanaka Y., Sugii Y., Kitamori T. (2011). Microchip-Based Plasma Separation from Whole Blood via Axial Migration of Blood Cells. Anal. Sci..

[17] Aran K., Fok A.A., Sasso L.A., Kamdar N., Guan Y., Sun Q., Ündar A., Zahn D.J. (2011). Microfiltration Platform for Continuous Blood Plasma Protein Extraction from Whole Blood during Cardiac Surgery. Lab Chip.

[18] Chen P.C., Chen C.C., Young K.C. (2016). Characterization of Thermoplastic Microfiltration Chip for the Separation of Blood Plasma from Human Blood. Biomicrofluidics.

[19] Liu C., Mauk M., Gross R., Bushman F.D., Edelstein P.H., Collman R.G., Bau H.H. (2013). Membrane-Based, Sedimentation-Assisted Plasma Separator for Point-of-Care Applications. Anal. Chem..

[20] Liu C., Liao S.C., Song J., Mauk M.G., Li X., Wu G., Ge D., Greenberg R.M., Yang S., Bau H.H. (2016). A High-Efficiency Superhydrophobic Plasma Separator. Lab Chip.

[21] Kadimisetty K., Yin K., Roche M.A., Yi Y., Bushman D.F., Collman G.R., Gross R., Feng L., Liu C. (2021). An Integrated Self-Powered 3D Printed Sample Concentrator for Highly Sensitive Molecular Detection of HIV in Whole Blood at the Point of Care. Analyst.

[22] Su X., Zhang S., Ge S., Chen M., Zhang J., Zhang J., Xia N. (2018). A Low Cost, Membranes Based Serum Separator Modular. Biomicrofluidics.

[23] Su X., Zhang J., Zhang D., Wang Y., Chen M., Weng Z., Wang J., Zeng J., Zhang Y., Zhang S. (2020). High-Efficiency Plasma Separator Based on Immunocapture and Filtration. Micromachines.

[24] Park C., Kim H.R., Kim S.K., Jeong I.K., Pyun J.C., Park S. (2019). Three-Dimensional Paper-Based Microfluidic Analytical Devices Integrated with a Plasma Separation Membrane for the Detection of Biomarkers in Whole Blood. ACS Appl. Mater. Interfaces.

[25] Wong A.P., Gupta M., Shevkoplyas S.S., Whitesides G.M. (2008). Egg Beater as Centrifuge: Isolating Human Blood Plasma from Whole Blood in Resource-Poor Settings. Lab Chip.

[26] Brown J., Theis L., Kerr L., Zakhidova N., O’Connor K., Uthman M., Oden Z.M., Richards-Kortum R. (2011). A Hand-Powered, Portable, Low-Cost Centrifuge for Diagnosing Anemia in Low-Resource Settings. Am. J. Trop. Med. Hyg..

[27] Bhamla M.S., Benson B., Chai C., Katsikis G., Johri A., Prakash M. (2017). Hand-Powered Ultralow-Cost Paper Centrifuge. Nat. Biomed. Eng..

[28] Allen W.M. (1950). A Simple Method for Analyzing Complicated Absorption Curves, of Use in the Colorimetric Determination of Urinary Steroids. J. Clin. Endocrinol. Metab..

[29] Cripps C.M. (1968). Rapid Method for the Estimation of Plasma Haemoglobin Levels. J. Clin. Pathol..

[30] Nader E., Skinner S., Romana M., Fort R., Lemonne N., Guillot N., Gauthier A., Antoine-Jonville S., Renoux C., Hardy-Dessources M.D. (2019). Blood Rheology: Key Parameters, Impact on Blood Flow, Role in Sickle Cell Disease and Effects of Exercise. Front. Physiol..

[31] Onódi Z., Pelyhe C., Terézia Nagy C., Brenner G.B., Almási L., Kittel A., Manček-Keber M., Ferdinandy P., Buzás E.I., Giricz Z. (2018). Isolation of High-Purity Extracellular Vesicles by the Combination of Iodixanol Density Gradient Ultracentrifugation and Bind-Elute Chromatography From Blood Plasma. Front. Physiol..

