# Supplementary material for: 3D Printed Devices for the Separation of Blood Plasma from Capillary Samples
Source: Micromachines (Basel). 2024 Feb 29;15(3):359. doi: 10.3390/mi15030359 (PMC10972198; doi:10.3390/mi15030359)
Supplement: Supplementary file 1 [file micromachines-15-00359-s001.zip › micromachines-2865130-supplementary.pdf]

Supplementary information

Design measurements

Please note that there is a significant difference between CAD and actual print measurements due to the relatively low quality that can be achieved with low-cost hobbyist 3D printers. A clear difference can be, for example, seen in the image below representing a device cross-section against one of the early designs. A trial and error approach was used to achieve optimal design parameters that would translate into a successful print.

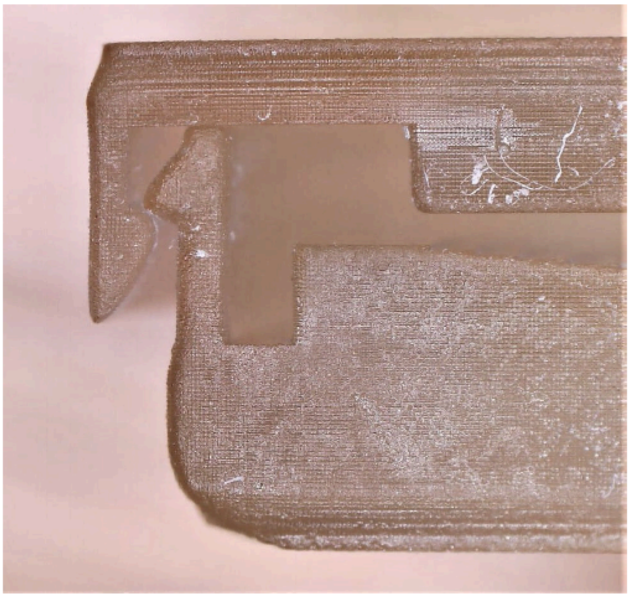

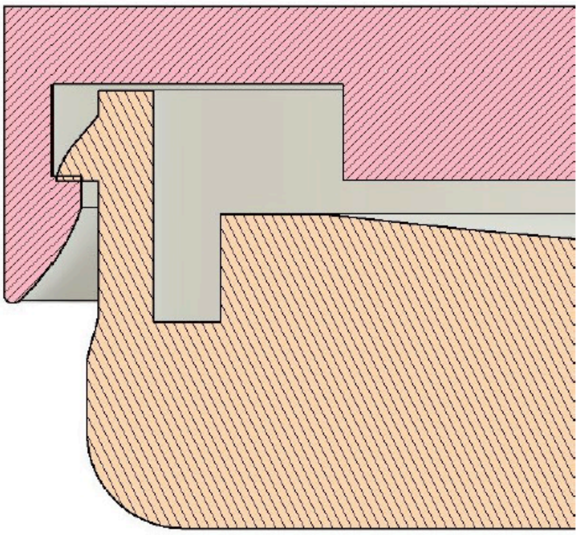

We offer here device drawings with the main measurements included and a full link to all CAD designs used in this study, plus a few extra containing the S2 device type with the patterns presented for the S1 devices.

Type B

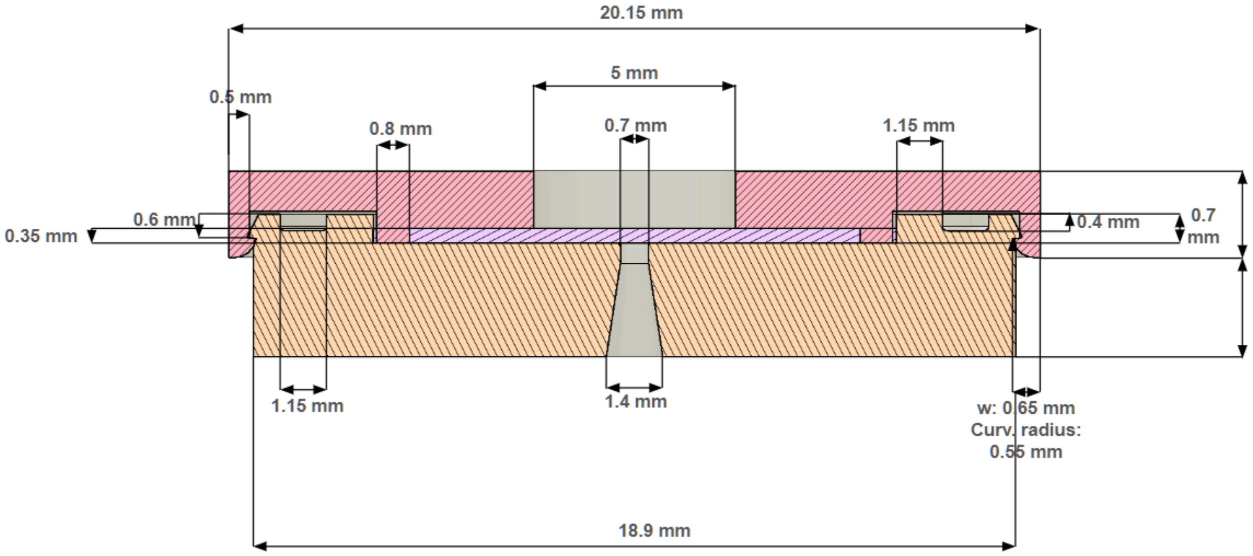

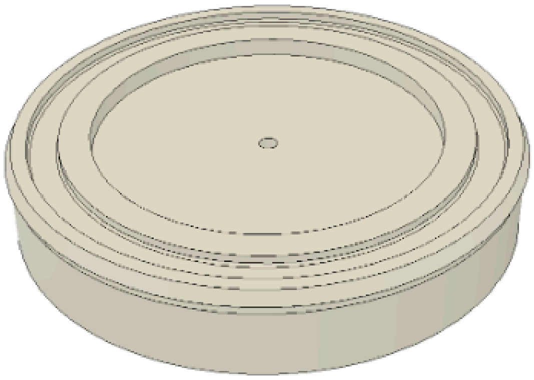

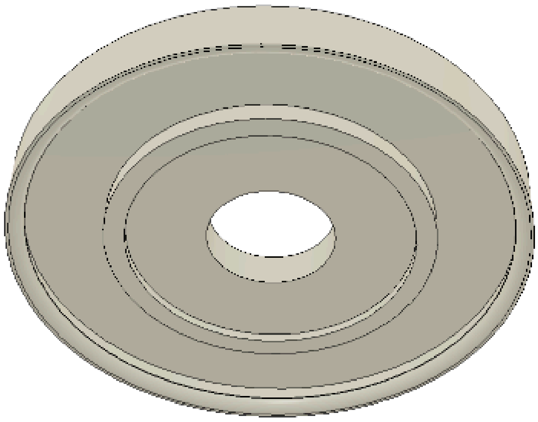

Type M

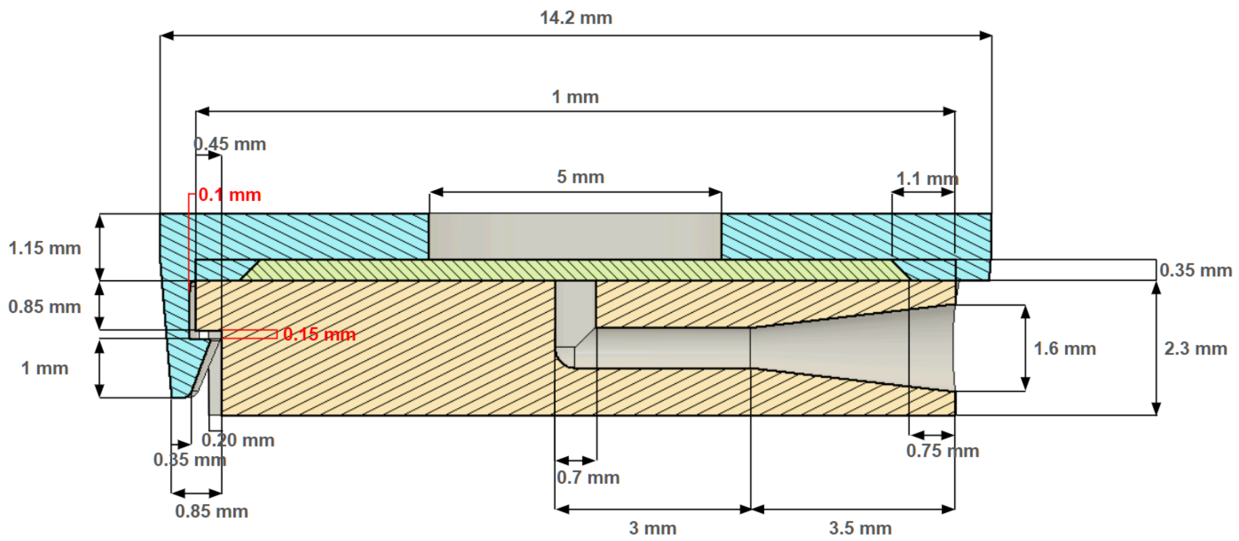

14.2 mm
1 mm
0.45 mm
5 mm
1.1 mm
0.1 mm
1.15 mm
0.35 mm
0.85 mm
0.15 mm
1 mm
1.6 mm
2.3 mm
0.20 mm
0.35 mm
0.75 mm
0.7 mm
0.85 mm
3 mm
3.5 mm

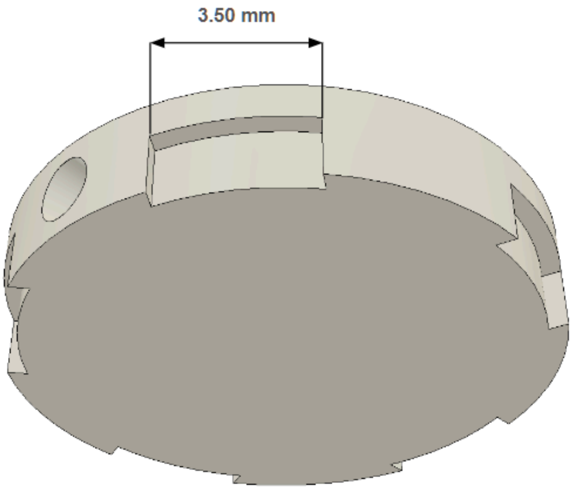

3.50 mm

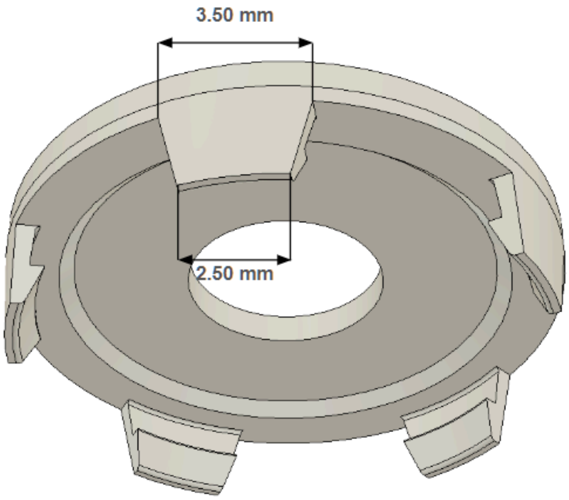

3.50 mm
2.50 mm

Type S1 and S2

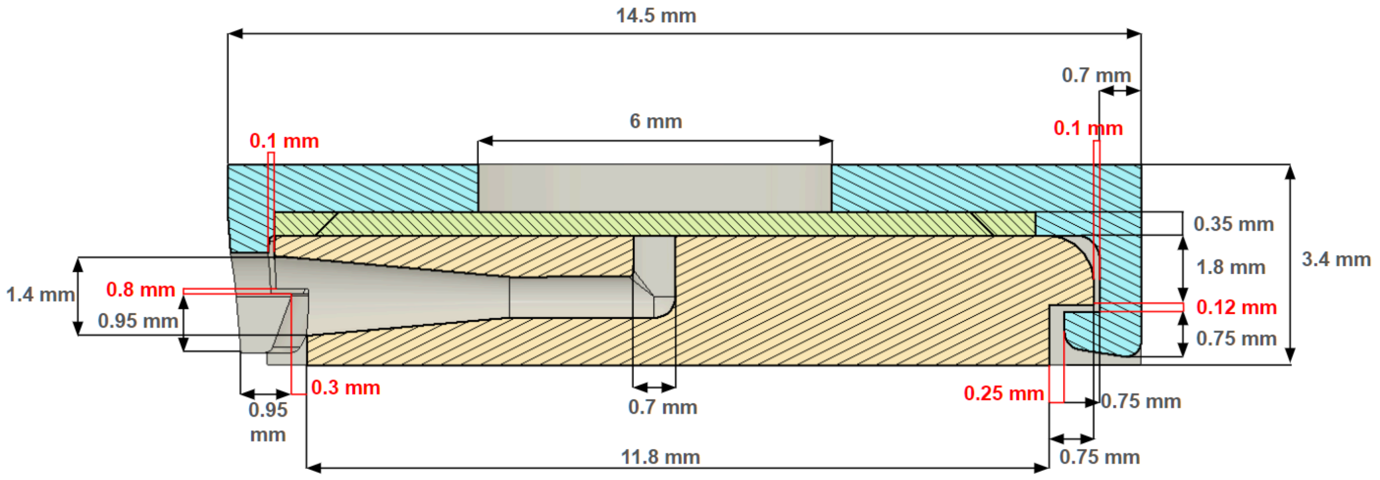

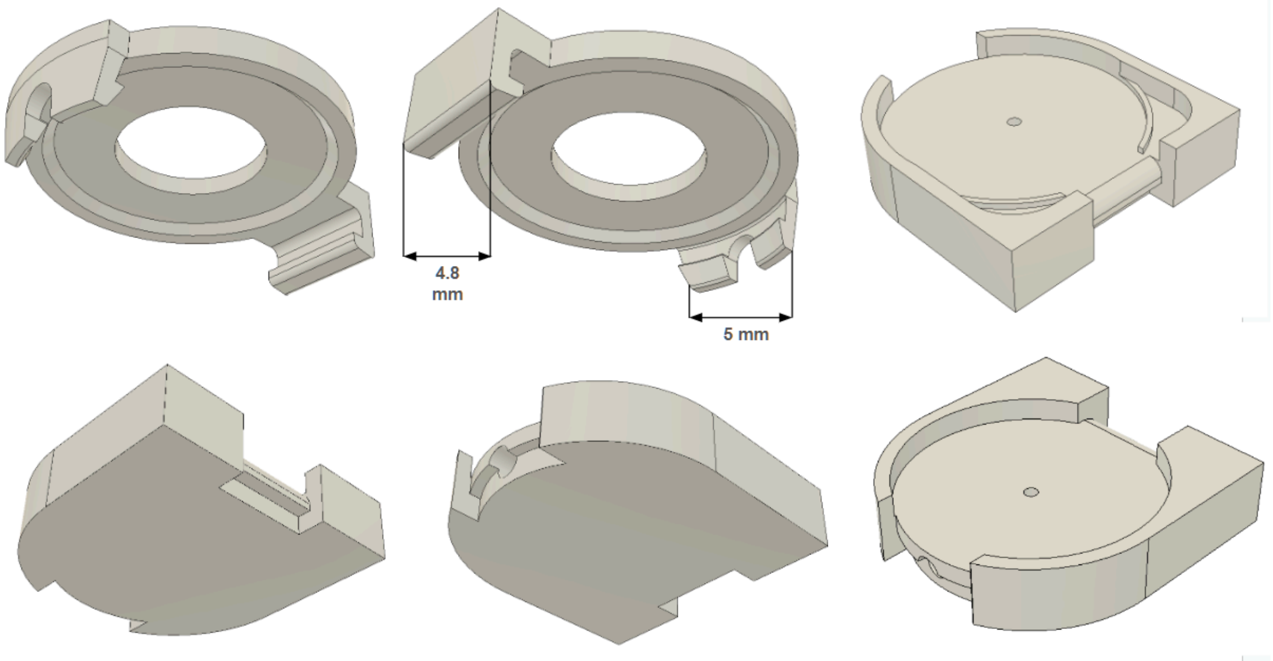

Design links

Type B - Bonded Design: https://a360.co/49r39Ay
Type M - Multi-Hook Design: https://a360.co/3SUDIG1
Type S1 - Single Hook V1: https://a360.co/4bRrTDK
Type S2 - Single Hook V2: https://a360.co/3uSCVYr
Pattern P1: https://a360.co/3SOOCaJ
Pattern P2: https://a360.co/3Ihv5KY
Pattern P3: https://a360.co/49pZP8Q
S1 Device with Pattern P1: https://a360.co/3SJJAw8
S1 Device with Pattern P2: https://a360.co/3IiWQ5T
S1 Device with Pattern P3: https://a360.co/3IcWIVO
S2 Device with Pattern P1: https://a360.co/3UNRBTt
S2 Device with Pattern P2: https://a360.co/3wtIixT S2
Device with Pattern P3: https://a360.co/3T9gdF0

Minimum channel depth

Microscope images of 3D printed channels with widths 50, 100, 140, 200, 240 and 300 µm at different orientations and magnification levels. The printing test was performed to determine the minimum possible channel width and was repeated for layer heights 0.025 mm and 0.05 mm.

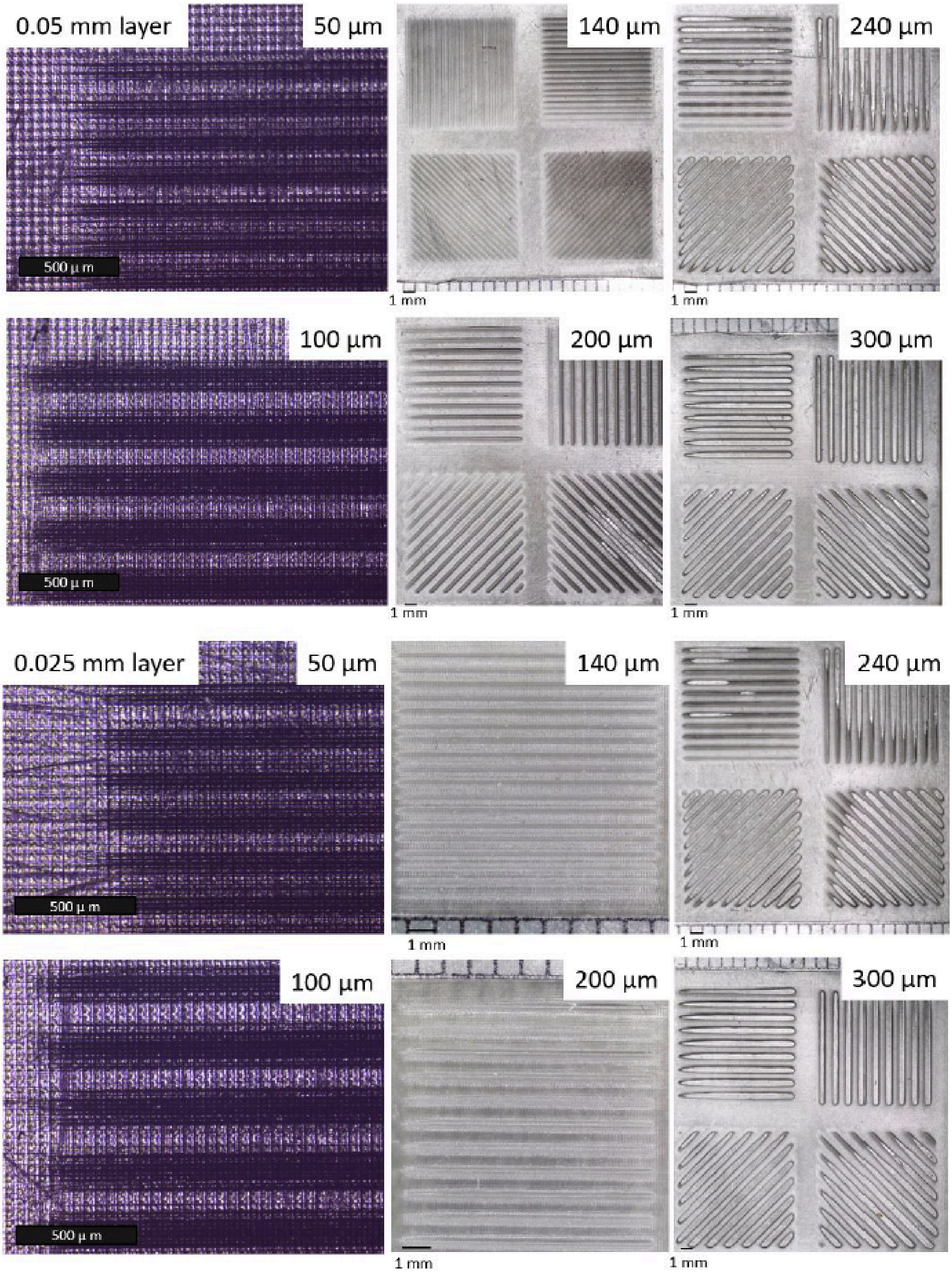

Preliminary tests for channel development

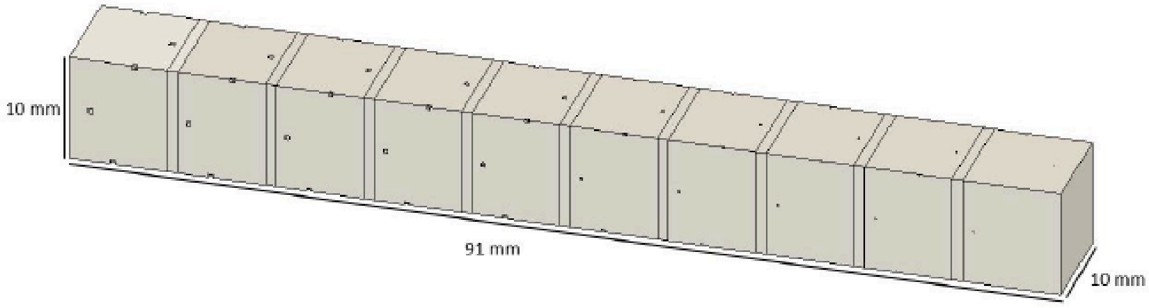

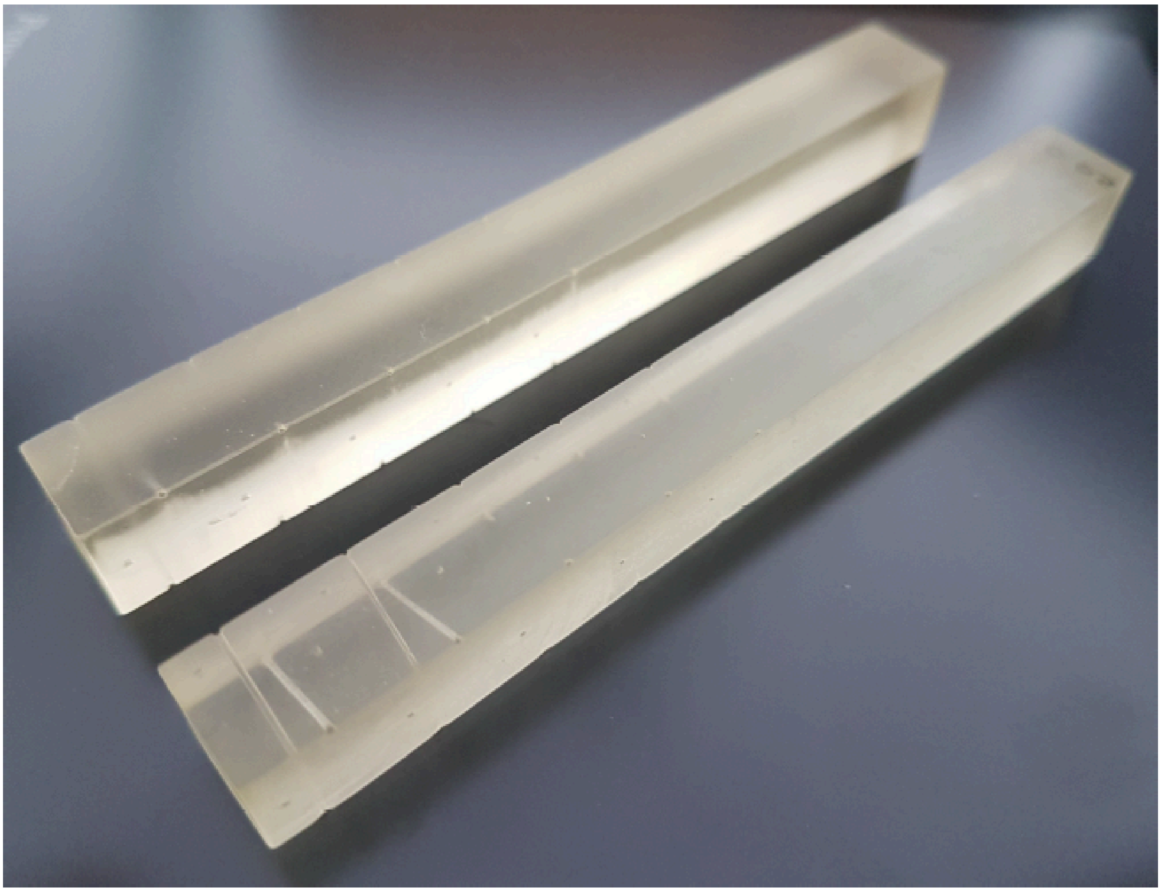

Top: design of the test part developed to observe the effect of orientation on the printing of internal channels. Bottom: corresponding printed parts. Diagonal orientations were the most successful, while at the same cross section the equivalent vertical and horizontal channel was clogged during printing.

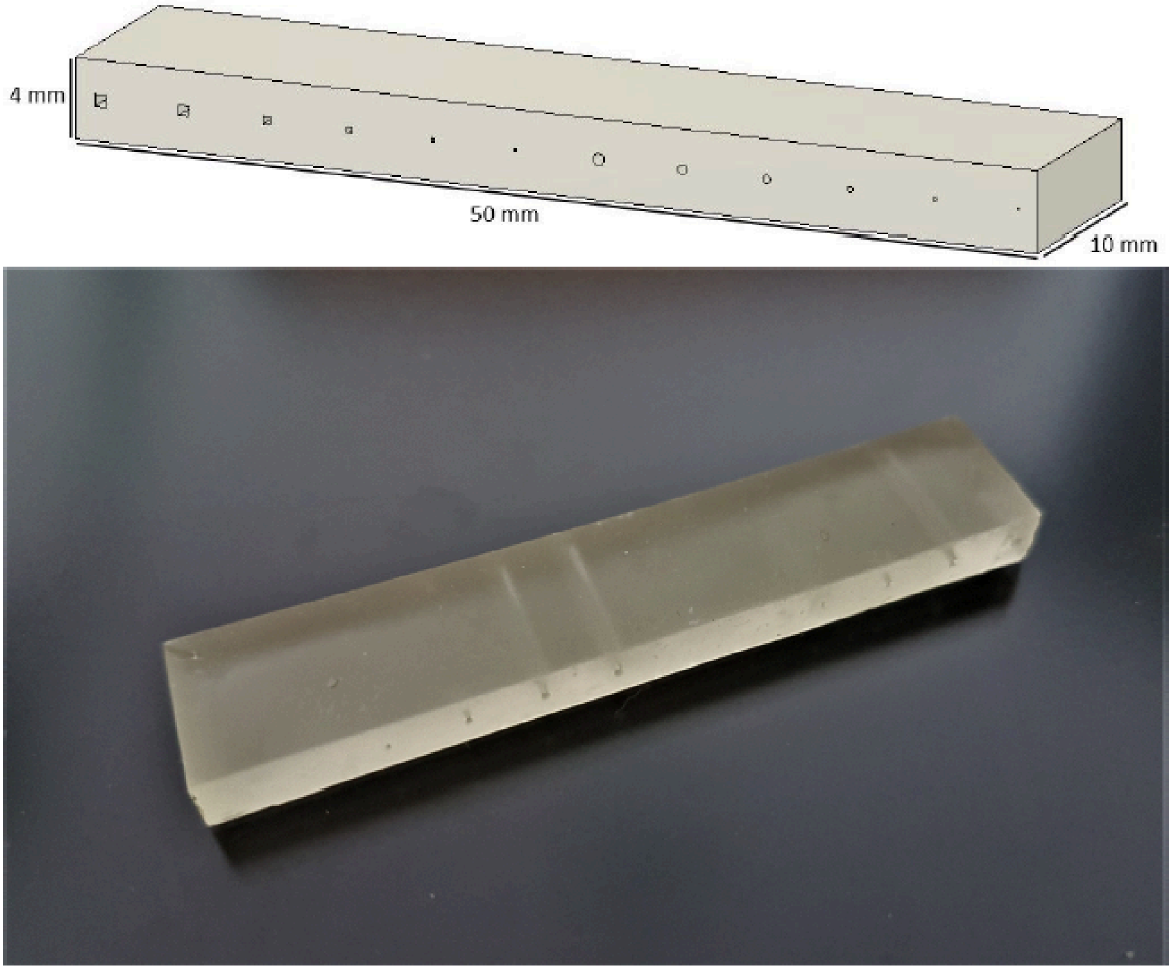

Top: design of the test part developed to determine the minimum internal channel width and diameter that can be printed with square and circular cross section respectively. Bottom: corresponding printed part. We tested circular cross sections of 0.6, 0.5, 0.4, 0.3, 0.2 and 0.1 mm diameter and square cross sections with dimensions 0.6 x0.6, 0.5 x0.5, 0.4 x0.4, 0.3x 0.3, 0.2 x0.2 and 0.1 x0.1 mm^2.

Only the circular 0.5 and 0.6 mm diameter and 0.6 mm width square channels were printed successfully, the rest were clogged during the printing process.
